# Supplementary material for: Photosynthesis-inspired H2 generation using a chlorophyll-loaded liposomal nanoplatform to detect and scavenge excess ROS
Source: Nat Commun. 2020 Jan 27;11:534. doi: 10.1038/s41467-020-14413-x (PMC6985250; doi:10.1038/s41467-020-14413-x)
Supplement: Supplementary file 1 — Supplementary Information [file 41467_2020_14413_MOESM1_ESM.pdf]

Supporting Information

Photosynthesis-Inspired H_2 Generation Using a Chlorophyll-Loaded Liposomal Nanoplatform to Detect and Scavenge Excess ROS

Wan et al.

Supplementary Table 1. Sizes of Lip NPs before and after Various Treatments (n = 6). Source data are provided as Source Data file.

Before Treatment	NIR Treatment	H_2O_2 Treatment	NIR+H_2O_2 Treatment
209.8 ± 12.3 nm	217.0 ± 15.3 nm	193.4 ± 9.3 nm	193.0 ± 16.3 nm

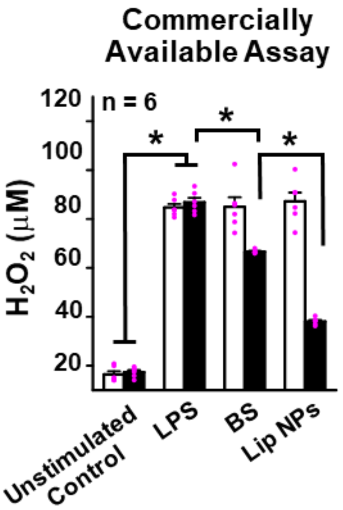

Supplementary Figure 1. Remaining H_2O_2 Concentrations. Concentrations of H_2O_2 that remained following various treatments, estimated by commercially available assay. Data are represented as mean ± SE. Stars indicate significance in the two-tailed unpaired Student's *t* test; *$P < 0.05$. Each pink dot represents one observed data point. Source data are provided as Source Data file.
