# Supplementary material for: Photosynthesis-inspired H2 generation using a chlorophyll-loaded liposomal nanoplatform to detect and scavenge excess ROS
Source: Nat Commun. 2020 Jan 27;11:534. doi: 10.1038/s41467-020-14413-x (PMC6985250; doi:10.1038/s41467-020-14413-x)
Supplement: Supplementary file 3 — Reporting Summary [file 41467_2020_14413_MOESM3_ESM.pdf]

nature research

Corresponding author(s): Hsing-Wen Sung

Last updated by author(s): Dec 13, 2019

Reporting Summary

Nature Research wishes to improve the reproducibility of the work that we publish. This form provides structure for consistency and transparency in reporting. For further information on Nature Research policies, see Authors & Referees and the Editorial Policy Checklist .

Statistics

For all statistical analyses, confirm that the following items are present in the figure legend, table legend, main text, or Methods section.

n/a	Confirmed	
☐	☒	The exact sample size (*n*) for each experimental group/condition, given as a discrete number and unit of measurement
☐	☒	A statement on whether measurements were taken from distinct samples or whether the same sample was measured repeatedly
☐	☒	The statistical test(s) used AND whether they are one- or two-sided<br>*Only common tests should be described solely by name; describe more complex techniques in the Methods section.*
☐	☒	A description of all covariates tested
☐	☒	A description of any assumptions or corrections, such as tests of normality and adjustment for multiple comparisons
☐	☒	A full description of the statistical parameters including central tendency (e.g. means) or other basic estimates (e.g. regression coefficient) AND variation (e.g. standard deviation) or associated estimates of uncertainty (e.g. confidence intervals)
☐	☒	For null hypothesis testing, the test statistic (e.g. *F*, *t*, *r*) with confidence intervals, effect sizes, degrees of freedom and *P* value noted<br>*Give P values as exact values whenever suitable.*
☒	☐	For Bayesian analysis, information on the choice of priors and Markov chain Monte Carlo settings
☐	☒	For hierarchical and complex designs, identification of the appropriate level for tests and full reporting of outcomes
☒	☐	Estimates of effect sizes (e.g. Cohen's *d*, Pearson's *r*), indicating how they were calculated

Our web collection on statistics for biologists contains articles on many of the points above.

Software and code

Policy information about availability of computer code

Data collection	Zetasizer Software, Zen 2 (blue edition), SoftMax Pro (6.3.1), FL Solutions (2514070-00), Lab Solutions
Data analysis	Excel 2016, and Origin 2016

For manuscripts utilizing custom algorithms or software that are central to the research but not yet described in published literature, software must be made available to editors/reviewers. We strongly encourage code deposition in a community repository (e.g. GitHub). See the Nature Research guidelines for submitting code & software for further information.

Data

Policy information about availability of data

All manuscripts must include a data availability statement. This statement should provide the following information, where applicable:

- Accession codes, unique identifiers, or web links for publicly available datasets
- A list of figures that have associated raw data
- A description of any restrictions on data availability

The authors declare that the data supporting the findings of this study are available within the paper.

Field-specific reporting

Please select the one below that is the best fit for your research. If you are not sure, read the appropriate sections before making your selection.

☒ Life sciences ☐ Behavioural & social sciences ☐ Ecological, evolutionary & environmental sciences

For a reference copy of the document with all sections, see nature.com/documents/nr-reporting-summary-flat.pdf

Life sciences study design

All studies must disclose on these points even when the disclosure is negative.

Sample size	No sample-size calculation was performed. The sample sizes for all quantification studies were n = 6, which were based on our previous publications (e.g., J Am Chem Soc. 2017,139,12923-12926).
Data exclusions	The outliers were excluded in a few experiments in the study. In general, the data with three standard deviations from the mean are regarded as outliers.
Replication	All attempts at replication were successful.
Randomization	We used the simple randomization method to allocate test samples into experimental groups.
Blinding	Blinding was not relevant to our study.

Reporting for specific materials, systems and methods

We require information from authors about some types of materials, experimental systems and methods used in many studies. Here, indicate whether each material, system or method listed is relevant to your study. If you are not sure if a list item applies to your research, read the appropriate section before selecting a response.

Materials & experimental systems

n/a	Involved in the study	
☐	☒	Antibodies
☐	☒	Eukaryotic cell lines
☒	☐	Palaeontology
☒	☐	Animals and other organisms
☒	☐	Human research participants
☒	☐	Clinical data

Methods

n/a	Involved in the study	
☒	☐	ChIP-seq
☒	☐	Flow cytometry
☒	☐	MRI-based neuroimaging

Antibodies

Antibodies used	DAPI (Invitrogen, D1306), IL-1b (abcam, ab9722), IL-6 (abcam, ab9324)
Validation	These are all commercially available antibodies. The use of these antibodies were based on the manufacturers' instructions.<br>https://www.thermofisher.com/order/catalog/product/D1306<br>https://www.abcam.com/il-1-beta-antibody-ab9722.html<br>https://www.abcam.com/il-6-antibody-ab9324.html

Eukaryotic cell lines

Policy information about cell lines

Cell line source(s)	The cell line (RAW264.7) used in the study was obtained from the Food Industry Research and Development Institute (FIRDI, Hsinchu, Taiwan). Its Bioresource Collection and Research Center is deemed the most comprehensive bioresource center in Asia (http://www.bcrc.firdi.org.tw/wwwbcrc/index.do).
Authentication	Please see the above statement.
Mycoplasma contamination	Confirm that all cell lines tested negative for mycoplasma contamination.
Commonly misidentified lines<br>(See ICLAC register)	The cell line used in this study was RAW264.7; it was obtained from FIRDI as stated above.
